# Supplementary material for: Conditional chemoconnectomics (cCCTomics) as a strategy for efficient and conditional targeting of chemical transmission
Source: eLife. 2024 Apr 30;12:RP91927. doi: 10.7554/eLife.91927 (PMC11060718; doi:10.7554/eLife.91927)
Supplement: Figure 3—source data 3. [file elife-91927-fig3-data3.zip › Figure 3 source data3 part 1.zip!/Figure 3-Source data 3-part1/1081-1-CRISPResso_Report_8zosk6yj.zip!/CRISPResso_on_1081_1/1c.Alignment_barplot.pdf]

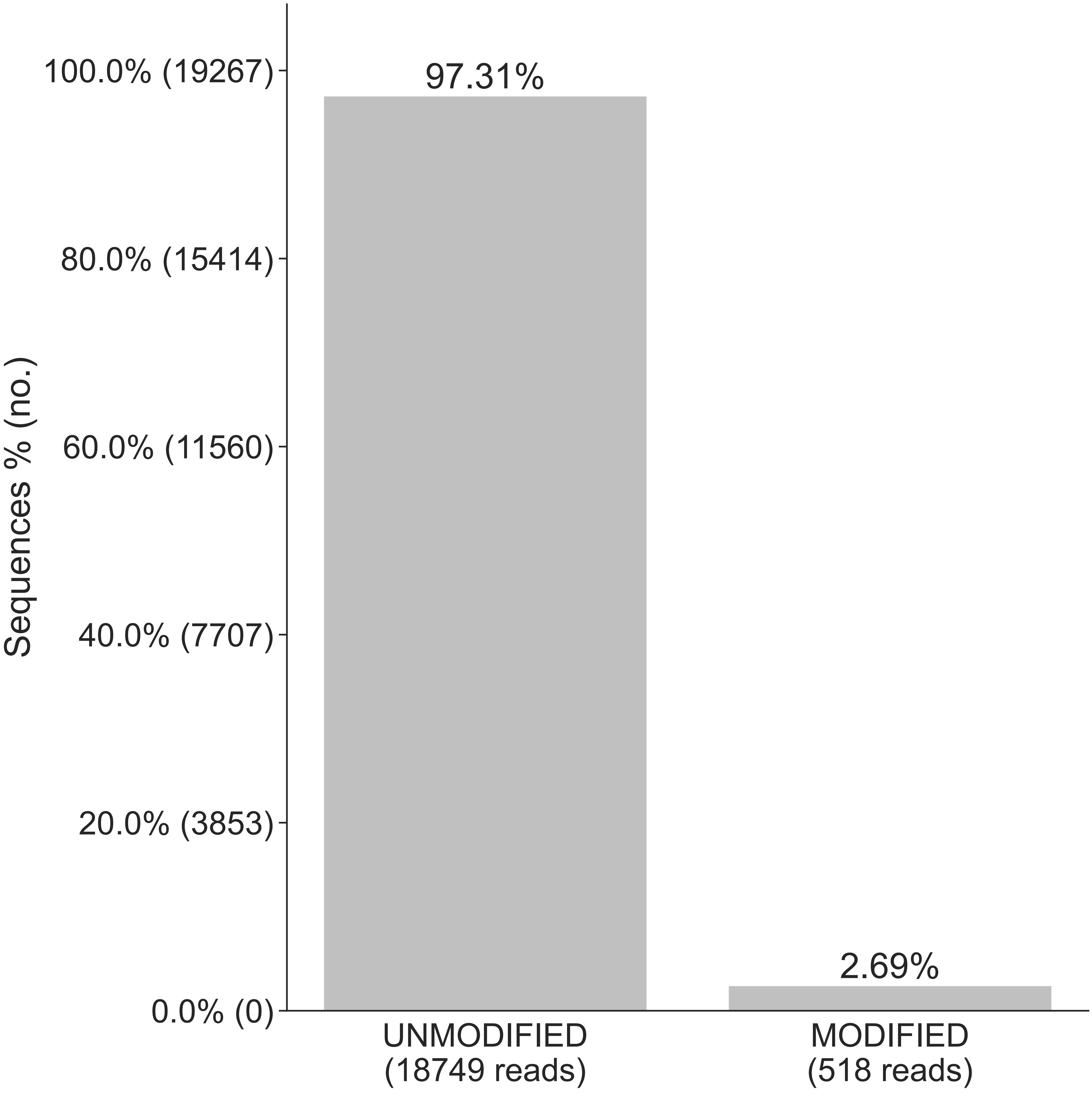
100.0% (19267)
80.0% (15414)
60.0% (11560)
40.0% (7707)
20.0% (3853)
0.0% (0)
Sequences % (no.)
97.31%
2.69%
UNMODIFIED
(18749 reads)
MODIFIED
(518 reads)
